# Incidence, Characteristics and Risk Factors of Acute Kidney Injury among Dengue Patients: A Retrospective Analysis

**DOI:** 10.1371/journal.pone.0138465

**Published:** 2015-09-30

**Authors:** Tauqeer Hussain Mallhi, Amer Hayat Khan, Azreen Syazril Adnan, Azmi Sarriff, Yusra Habib Khan, Fauziah Jummaat

**Affiliations:** 1 Discipline of Clinical Pharmacy, School of Pharmaceutical Sciences, University Sains Malaysia, Penang, Malaysia; 2 Chronic Kidney Disease Resource Centre, School of Medical Sciences, Health Campus, University Sains Malaysia, Kubang Kerain, Kelantan, Malaysia; 3 Department of Obstetrics and Gynecology, School of Medical Sciences, Health Campus, University Sains Malaysia, Kubang Kerain, Kelantan, Malaysia; Bambino Gesù Children's Hospital, ITALY

## Abstract

**Background:**

Dengue induced acute kidney injury (AKI) imposes heavy burden of illness in terms of morbidity and mortality. A retrospective study was conducted to investigate incidence, characteristics, risk factors and clinical outcomes of AKI among dengue patients.

**Methodology:**

A total 667 dengue patients (2008–2013) were retrospectively evaluated and were stratified into AKI and non-AKI groups by using AKIN criteria. Two groups were compared by using appropriate statistical methods.

**Results:**

There were 95 patients (14.2%) who had AKI, with AKIN-I, AKIN-II and AKIN-III in 76.8%, 16.8% and 6.4% patients, respectively. Significant differences (*P*<0.05) in demographics and clinico-laboratory characteristics were observed between patients with and without AKI. Presence of dengue hemorrhagic fever [OR (95% CI): 8.0 (3.64–17.59), *P*<0.001], rhabdomyolysis [OR (95% CI): 7.9 (3.04–20.49)], multiple organ dysfunction [OR (95% CI): 34.6 (14.14–84.73), *P*<0.001], diabetes mellitus [OR (95% CI): 4.7 (1.12–19.86), *P* = 0.034], late hospitalization [OR (95% CI): 2.1 (1.12–19.86), *P* = 0.033] and use of nephrotoxic drugs [OR (95% CI): 2.9 (1.12–19.86), *P* = 0.006] were associated with AKI. Longer hospital stay (>3 days) was also observed among AKI patients (OR = 1.3, *P* = 0.044). Additionally, 48.4% AKI patients had renal insufficiencies at discharge that were signicantly associated with severe dengue, secondary infection and diabetes mellitus. Overall mortality was 1.2% and all fatal cases had AKI.

**Conclusions:**

The incidence of AKI is high at 14.2% among dengue patients, and those with AKI portended significant morbidity, mortality, longer hospital stay and poor renal outcomes. Our findings suggest that AKI in dengue is likely to increase healthcare burden that underscores the need of clinicians’ alertness to this highly morbid and potentially fatal complication for optimal prevention and management.

## Introduction

Dengue viral infection (DVI) is a mosquito borne disease that imperils 20 million people every year in tropical and subtropical regions [1]. Currently more than 40% world population is at risk of being infected by dengue virus [2]. DVI is manifested with variety of clinical presentations including asymptomatic infection, undifferentiated fever, dengue fever (DF), dengue hemorrhagic fever (DHF) and life threatening dengue shock syndrome (DSS). Like other tropical infections, DVI is associated with multiple organ dysfunction [3] effecting liver, muscles, heart, brain and kidneys [4]. Spectrum of renal disorders is least studied in dengue infection that varies from mild glomerulonephritis, urinary sedimentations to severe acute kidney injury (AKI) [5].

AKI is a rare complication of DVI and is associated with poor prognosis. Previous investigations have shown great disparity in incidence of dengue induced AKI ranging from 0.83% to 13.3% [6–14]. Similarly AKI associated mortality among dengue patients varies from 11.3% to 60% [6, 11]. In most of the previously conducted studies, AKI was evaluated among severe dengue cases i.e. DHF [9, 11] while occurrence of AKI in DF has also been reported by few case reports [15–22]. There is still scarcity of data and we found only two small case series [6, 7] investigating AKI by including all types of dengue infection, irrespective of severity. Therefore thorough investigation is urgently required to reveal clinico-laboratory characteristics, and, more importantly, risk factors of this complication to reduce associated morbidity and mortality.

According to WHO, Malaysia is the only Asian country where incidence of DVI is rapidly escalating. Approximately 84, 682 cases of DVI and 160 associated deaths have been reported to WHO till 20 December 2014 that are substantially higher as compared to same period in 2013 [23]. However, data on epidemiology and outcomes concerning dengue induced AKI in Malaysia has not been studied. Therefore, we took an opportunity and a retrospective case series study was conducted to determine incidence, characteristics, risk factors and discharge outcomes of AKI among dengue patients.

## Methodology

Current study was conducted in Hospital University Sains Malaysia (HUSM), tertiary level teaching hospital with 950 beds that serves an estimated 1.4 to 1.8 million inhabitants of Kelantan. Kelantan is an agrarian state located in the north-east of Peninsular Malaysia and among top five dengue hotspots in the country where the dengue cases are substantially rising every year. Malays are major (95%) ethnic group in Kelantan while Chinese constitutes merely 4% of state population. The hospital also serves as referral centers for nearby states.

**Fig 1:**
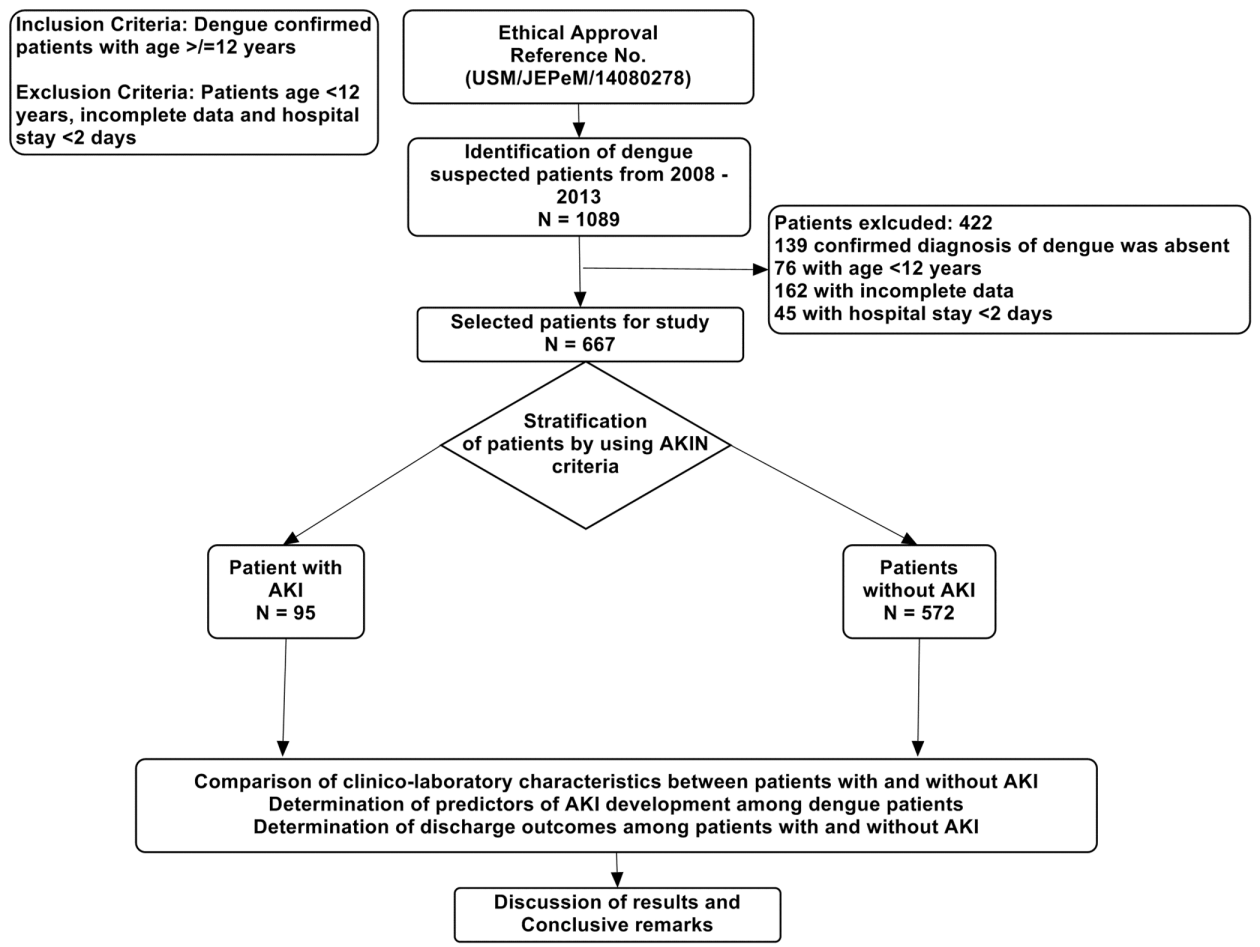
Study Methodology Flow Diagram.

We retrospectively reviewed medical records of all dengue patients admitted from January 2008 to December 2013. All dengue patients were identified by registration number using hospital record management system. Patients having age ≥ 12 years admitted with primary and confirmed diagnosis of DVI, irrespective of severity, were included in this study. Methodology of study flow is given in Fig 1. Patients having incomplete demographics and hospital stay less than 2 days were excluded from the study. Suspected DVI cases were diagnosed by using at least one of the following criteria: (1) positive reverse transcriptase polymerase chain reaction (RT-PCR) result, (2) presence of dengue immunoglobulin M and G antibodies in acute phase serum by enzyme linked immunosorbent assay [Pan Bio Dengue IgM ELISA, Dengue IgM Dot Enzyme Immunoassay, SD Dengue IgM and IgG capture ELISA Kits; Standard Diagnostics, Korea], and (3) at least 4-fold increase of dengue-specific hemagglutination inhibition titers in convalescent serum when compared with acute phase serum. The serum samples were also tested for dengue-specific NS1 [pan-E Early dengue ELISA kit by Panbio, Australia and Platelia dengue NS1Ag assay by Bio-Rad Laboratories, USA). Primary dengue infection was distinguished from secondary infection by using IgM/IgG ratio where dengue infection was defined as primary if ratio > 1.2 and as secondary if < 1.2 [24] or if there was a 4-fold increase of HAI and the titers were ≤1:1280 and ≥1:2560, respectively [9]. Serologically confirmed dengue patients were subjected to clinical case definition and disease severity was classified according to the WHO criteria, where clinical diagnosis of DF requires fever and two or more of following symptoms; headache, retro-orbital pain, myalgia, arthralgia, rash, hemorrhagic manifestations and leucopenia; DHF requires presence of fever, thrombocytopenia (≤100 × 10^9^/L), any bleeding and plasma leakage described as either hematocrit change ≥20%, clinical fluid accumulation (pleural effusion or ascites), or hypoproteinemia; and DSS requires presence of one of rapid and weak pulse with narrow pulse pressure <20 mmHg or hypotension for age in a patient with DHF. Presence of warning signs indicates the presence of at least one of the following; abdominal pain/tenderness, persistent vomiting, clinical fluid accumulation, mucosal bleed, lethargy/restlessness, liver enlargement> 2 centimeter, concurrent increase in hematocrit with thrombocytopenia [25].

AKI was defined by Acute Kidney Injury Network (AKIN) classification [26]. Patients without baseline SCr and having no history of chronic renal insufficiency (CRI), baseline SCr was estimated with Modification of Diet in Renal Disease (MDRD) equation by assuming glomerular filtration rate as 75 ml/min/1.73m^2^. Approximately 62% of all patients were classified with estimated SCr. About 4.9% patients in our study had CKD and all of them were on regular follow-up in our institution. For such patients’ baseline SCr level was determined as the lowest value among SCr levels during their preceding 3 months before infection. Patients were staged according to SCr or urine output criteria and this comprised criteria that led to the worst possible classification during hospital stay.

All patient`s demographics, clinical and laboratory data were recorded by using structured case report form. Demographics and clinical presentations were noted at hospital admission while laboratory data was noted for each day of hospitalization. Hospital computerized record system and patients’ files were evaluated for the purpose of data collection. Patients were evaluated for discharge outcomes i.e. fully recovered (without renal and hepatic impairments); normal renal function (without evidence of SCr elevation and renal deterioration), renal insufficiency with Scr <200 μmol/L, renal insufficiency with Scr >200 μmol/L, normal hepatic functions (normal ALT and AST levels), mild elevated transaminases (2–10 × upper limit normal), moderate to severe elevated transaminases (>10 × upper limit normal) and mortality. In order to discharge outcomes reference values for AST and ALT were 5–34 IU/L and 10–35 IU/L.

### Definitions

For the purpose of current study, terms used were defined as follow:

Hospital stay is defined by >1 day bed occupancy in hospital; mortality means death within 14 days after admission; hypokalemia (K < 3.5 mmol/L); hyponatremia (Na < 135 mmol/L); oliguria (UO < 400 ml/day after 24 hours of appropriate hydration); hypotension (blood pressure < 110/70 mmHg); elevated transaminases (elevation of liver enzymes such as aspartate aminotransferase [AST] and/or alanine aminotransferase [ALT] >2 times the normal value); transaminitis (elevation of both ALT and AST), prolonged prothrombin time (PT > 15 seconds); prolonged activated partial thromboplastin time (aPTT > 35 seconds); urinary sedimentations (presence of glycosuria, hematuria, proteinuria, leucocytouria, urine pus, urine epithelial cells); anemia (Hb < 12 g/dL); dengue viral infection (dengue fever, dengue hemorrhagic fever, dengue shock syndrome); severe dengue (DHF, DSS); mild AKI (AKIN-I); severe AKI (AKIN-II, AKIN-III); transaminitis (elevation of both ALT and AST); multiple organ dysfunction (dysfunction of ≥2 organs, including AKI); hepatic dysfunction (elevation of liver enzymes); and thrombocytopenia (platelets count < 100×10^9^ cells). Reference values for different laboratory parameters in current study are according to hospital laboratory i.e. calcium (2.2–2.5 mmol/L); serum phosphorous (0.9–1.3 mmol/L); serum chloride (95–105 mmol/L); BUN (1.7–8.3 mmol/L); uric acid (210–420 μmol/L); albumin (38–44 g/L); AST (5–34 IU/L); ALT (10–35 IU/L); ALP (♂: 53–168 ♀: 42–98 IU/L); total bilirubin (<17 μmol/L); BUN/creatinine ratio (5–35); urine specific gravity (1.005–1.030); urinary pH (4.7–7.8); creatine kinase (♂: ≤171 ♀: ≤145 IU/L).

### Ethics Statement

Permission (Reference No. USM/JEPeM/14080278) to conduct current study was obtained from ethical review board [Jawatankuasa Etika Penyelidikan (Manusia) of USM (JEPeM)] of Hospital University Sains Malaysia (HUSM). Personally identifiable information of patients was encrypted and all the analyzed data were anonymized.

### Statistical Analysis

Statistical analyses were done with SPSS 20.0 for Windows (SPSS Inc., USA). Quantitative variables were expressed as mean ± SD while qualitative data were presented as number of observations with percentages. Continuous data was compared by using Student *t*-test or Mann-Whitney *U* test, where appropriate. For the comparison of categorical or dichotomous data, Fisher exact test or *X* ^*2*^ test were used. *Phi* coefficient and Cramer`s *V* were used to evaluate strength of association between 2 by 2 or greater than 2 by 2 variables respectively. The association was referred weak, moderate or strong if *Phi* coefficient and Cramer’s *V* values were (0.1–0.29), (0.3–0.49) and (0.5–1.0) respectively. A logistic regression model was performed to determine factors independently associated with AKI. Variables with *P*-value <0.25 in univariate analysis were subjected to multivariate regression model [27]. Adjusted OR and 95% confidence interval were calculated. A double-sided *P* value <0.05 was considered statistically significant.

## Results

A total 1089 patients with suspected DVI were enrolled in current study and 422 patients were excluded (139 patients don’t have confirmed diagnosis of DVI; 76 patients with age < 12 years; 162 patients with incomplete demographics and laboratory data; 45 patients having hospital stay less than 2 days). Finally 667 patients were included in study for analysis.

### Clinical and Epidemiological characteristics of study cohorts

During the 6-year study period, 667 patients aged >12 years [mean age: 30.68 ± 16.13 years], needed hospitalization due to clinically diagnosed DVI. Of these, 95 (14.2%) patients developed AKI according to AKIN classification. Based upon severity, AKIN-I was observed in 76.8% while AKIN-II and AKIN-III were present in 16.8% and 6.4% patients, respectively. A total 84 (12.6%) patients had developed AKI prior to the admission with AKIN-I in 69, AKIN-II in 12 and AKIN-III in 4 cases while only 11 (1.6%) patients developed AKI after admission with AKIN-I in 5, AKIN-II in 4 and AKIN-III in 2 cases. We used SCr levels as criteria to define AKI due to missing data of UO for each day of hospitalization. But with available data, UO criteria classified 61(9.2%) patients into AKI and all of them were also stratified by SCr criteria, in addition to 34 (5%) new cases. Out of 84 patients with AKI on hospital admission, six patients with AKIN-I progressed to AKIN-II and three patients with AKIN-II progressed to AKIN-III while no patient with AKIN-I was progressed to AKIN-III. Similarly, out of 11 patients who developed AKI during hospitalization, 2 patients with AKIN-I progressed to AKIN-II and one of them further progressed to AKIN-III. The most severe degree of AKI was recorded in these cases. DHF and DSS were presented at admission and observed in 36% and 3% of AKI cases, respectively. None of the patient with DF progressed to DHF or DSS during hospitalization. Out of 74 patients with DHF and 5 patients with DSS, AKI was observed in 34 (46%) and 3 (60%) cases, respectively. Table 1 and Table 2 demonstrate comparative analysis of phenotype of patients including clinical and laboratory characteristics among dengue patients with and without AKI. We found significant differences in age (*P* = 0.008), gender (*P* < 0.001), dengue classification (*P* < 0.001), underline diseases (*P* < 0.001), secondary infection (*P* = 0.003), presence of warning signs (*P* = 0.020), transaminitis (*P* = 0.005), rhabdomyolysis (*P* = 0.008) and urinary sedimentations (*P* < 0.05). Out of total AKI cases, hypokalemia was observed in 21.1% patients while oliguria was present in 50.5% patients. The incidence of hypokalemic-oliguric AKI was 9.5%. Co-morbid diseases e.g. diabetes mellitus (*P* < 0.001), hypertension (*P* < 0.001), ischemic heart disease (*P* < 0.001) and hyperlipidemia (*P* = 0.010) were more significantly found in patients with AKI (Table 1).

**Table 1:** Comparison of patients with DVI with and without AKI.

Parameters	Overall patients N = 667	AKI group N = 95	Non-AKI group N = 572	*P*-value*
**Age groups**				0.008^1^ **
Adults (12–60 years)	639 (95.8%)	86 (90.5%)	553 (96.7%)	
Elders (> 60 years)	28 (4.2%)	9 (9.5%)	19 (3.3%)	
**Gender**				<0.001^1^ **
Male, n (%)	378 (56.7%)	71 (74.3%)	307 (53.7%)	
Female, n (%)	289 (43.3%)	24 (25.7%)	265 (46.3%)	
**Resident**			.	0.422
Urban	403 (60.4%)	54 (43.2%)	349 (61%)	
Rural	264 (39.6)	41 (56.8%)	223 (39%)	
**Diagnosis**				<0.001^4^ **
DF, n (%)	588 (88.6%)	58 (61%)	530 (96.2%)	<0.001**
DHF, n (%)	74 (10.6%)	34 (35.8%)	40 (7%)	<0.001**
DSS, n (%)	5 (0.7%)	3 (3.16%)	2 (0.35%)	0.005**
Presence of warning signs, n (%)	271 (40.6%)	49 (51.6%)	222 (38.8%)	0.020^1^ **
**Onset of dengue infection**				0.003^1^ **
Primary, n (%)	594 (89.1%)	76 (80%)	518 (90.5%)	
Secondary, n (%)	73 (10.9%)	19 (20%)	54 (9.5%)	
**Pre-Morbid Diseases**	119 (17.8%)	34 (35.8%)	85 (14.9%)	<0.001^4^ **
Diabetes mellitus, n (%)	36 (5.4%)	21 (22.1%)	15 (2.6%)	<0.001**
Hypertension, n (%)	35 (5.2%)	15 (15.8%)	20(3.5%)	<0.001**
CKD, n (%)	33 (4.9%)	7 (7.4%)	26 (4.5%)	0.245
IHD, n (%)	25 (3.7%)	13 (13.7%)	12 (2.1%)	<0.001**
CHF, n (%)	2 (0.3%)	0 (0%)	2 (0.4%)	0.999
HPL, n (%)	8 (1.2%)	4 (1.05%)	4 (0.7%)	0.01**
Temperature > 38 C°	220 (33%)	31 (32.6%)	189 (33%)	0.706
Co-infection, n (%)	55 (8.2%)	8 (8.4%)	47 (8.25%)	0.947
Hospitalization > 3 days, n (%)	338 (49.2%)	62 (65.3%)	276 (48.3%)	0.002**
Use of nephrotoxic drugs, n (%)	112 (16.8%)	50 (52.6%)	62 (10.8%)	<0.001**
Need of blood transfusion, n (%)	39 (5.8%)	7 (7.4%)	32 (5.6%)	0.496
Hypokalemia, n (%)	169 (25.3%)	20 (21%)	139 (24.3%)	0.309
Hyponatremia, n (%)	258 (38.7%)	43 (45.3%)	215 (37.6%)	0.175
Oliguria, n (%)	295 (44.2%)	48 (50.5%)	247 (43.2%)	0.186
Thrombocytopenia, n (%)	394 (59.1%)	62 (65.3%)	332 (58%)	0.217
Transaminitis, n (%)	360 (54%)	63 (66.3%)	297 (51.9%)	0.005^1^ **
Respiratory failure, n (%)	11 (1.6%)	2 (2.1%)	9 (1.6%)	0.707
Rhabdomyolysis, n (%)	49 (7.4%)	34 (35.8%)	15 (2.62%)	<0.001^2^ **
Multiple organ dysfunctions, n (%)	93 (14%)	82 (86.3%)	11 (2%)	<0.001^3^ **
Prolonged PT, n (%)	229 (33.6%)	43 (45.3%)	186 (32.5%)	0.015**
Prolonged aPTT, n (%)	159 (23.8%)	20 (21%)	139 (24.3%)	0.128
Urinary Sedimentations				
Proteinuria, n (%)	88 (13.2%)	26 (27.4%)	62 (10.8%)	<0.001^1^ **
Hematuria, n (%)	12 (1.8%)	6 (6.3%)	6 (1%)	0.002^1^ **
Glycosuria, n (%)	29 (4.3%)	8 (8.4%)	21 (3.7%)	0.041**
Urine pus, n (%)	23 (3.4%)	9 (9.5%)	14 (2.4%)	0.001^1^ **
Urinary epithelia cells, n (%)	15 (2.2%)	8 (8.4%)	7 (1.2%)	<0.001^1^ **
Mortality, n (%)	8 (1.2%)	8 (8.4%)	0 (0%)	<0.001**

Multiple organ dysfunctions is referred as dysfunction of ≥2 organ systems; DF: dengue fever, DHF: dengue hemorrhagic fever, DSS: dengue shock syndrome; CKD: chronic kidney disease, IHD: ischemic heart disease, DM: diabetes mellitus, CHF: congestive heart failure; HPL: hyperlipidemia, PT: prothrombin time, aPTT: activated partial thromboplastin time

*Fisher exact test, student *t* test or Mann-Whitney *U* test, where appropriate

**Significant

^1^Phi coefficient (0.1–0.29)

^2^Phi coefficient (0.3–0.49)

^3^Phi coefficient (0.5–1.0)

^4^Cramer`s V coefficient (0.3–0.49)

**Table 2:** Comparison of laboratory characteristics among DVI patients with and without AKI.

Parameters	Overall patients N = 667	AKI group N = 95	Non-AKI group N = 572	*P*-value
Age (years, mean ± SD)	30.68 ± 16.13	40.75 ±17.24	29.01 ±15.32	<0.001*
Anthropometric Characteristics				
Weight (kg, mean ± SD)	56.84 ±17.71	66.36 ± 13.2	55.62 ±17.86	<0.001*
Height (cm, mean ± SD)	151.78 ±18.06	160.26 ± 16.51	150.58 ±17.97.	0.001*
BMI (kg/m^2^, mean ± SD)	24..42 ± 6.46	25.67 ± 4.06	24.24 ± 6.72.	0.054
Serum creatinine (μmol/L, mean ± SD)	99.13 ± 58.49	175.17 ± 124.35	86.50 ± 17.99	<0.001*
BUN (mmol/L, mean ± SD)	4.44 ± 3.43	7.73 ± 7.35	3.89 ± 1.63	<0.001*
BUN: Cr ratio	11.58 ± 6.06	12.31 ± 11.18	11.46 ± 4.69	0.215
Uric acid (μmol/L, mean ± SD)	296.34 ± 112.18	371.34 ± 145.81	282.16 ± 98.59	<0.001*
Serum K (mmol/L, mean ± SD)	3.74 ±.55	3.83 ±.59	3.73 ± 0.54	0.112
Serum Na (mmol/L, mean ± SD)	135.23 ± 7.12	134.95 ± 6.18	135.28 ± 7.27	0.679
Serum albumin (g/L, mean ± SD)	40.26 ± 15.35	39.05 ± 5.89	40.48 ± 16.49	0.428
AG ratio	1.79 ± 4.46	1.49 ±.47	1.84 ± 4.8	0.113
AST (IU/L, mean ± SD)	152.42 ± 209.75	188.68 ± 256.79	145.86 ± 199.69	0.083
ALT (IU/L, mean ± SD)	114.36 ± 166.64	171.74 ± 236.41	104.40 ± 149.43	0.015*
ALP (IU/L, mean ± SD)	105.85 ± 71.32	110.11 ± 73.02	105.11 ± 71.08	0.558
Total bilirubin (μmol/L, mean ± SD)	12.29 ± 18.39	14.60 ± 18.64	11.86 ± 18.34	0.207
Urine specific gravity (mean ± SD)	1.012 ±.009	1.012 ±.011	1.013 ±.009	0.801
Urinary pH (mean ± SD)	6.2 ± 1.6	6.53 ±.862	6.17 ± 1.74	0.177
Urine volume (ml/24 hours, mean ± SD)	988.4 ± 692.5	982.37 ± 670.37	989.4 ± 696.8	0.932
Creatinine kinase (IU/L, mean ± SD)	234.61 ± 125.2	300.4 ± 145.4	152.4 ± 89.3	0.002*
Leucocytes (×10^9^/L, mean ± SD)	5.15 ± 14.77	5.30 ± 4.35	5.13 ± 15.83	0.918
Erythrocytes (×10^12^/L, mean ± SD)	4.92 ± 0.63	4.88 ±.51	4.92 ± 0.65	0.105
Thrombocytes (×10^9^/L, mean ± SD)	97.59 ± 63.14	89.51 ± 48.29	98.89 ± 65.15	0.196
Hemoglobin (g/dL, mean ± SD)	14.21 ± 7.97	14.38 ± 1.63	14.18 ± 8.54	0.631
Hematocrit (%/L, mean ± SD)	40.18 ± 5.09	41.49 ± 4.47	39.98 ± 5.16	0.005*
PT (seconds, mean ± SD)	13.13 ± 1.35	15.42 ± 2.84	11.84 ± 0.88	<0.001*
aPTT (seconds, mean ± SD)	44.23 ± 9.01	44.74 ± 7.99	44.15 ± 9.18	0.563
International normalized ratio (INR)	1.05 ± 0.56	1.17 ±.96	1.0329 ± 0.44	0.227
Length of hospitalization (days, mean ± SD)	4.87 ± 2.75	5.79 ± 3.45	4.72 ± 2.58	0.005*
Period of illness prior to hospitalization (days, mean ± SD)	4.16 ± 3.28	5.89 ± 1.21	3.72 ± 2.34	<0.001*

*Significant, *P* value is subjected to difference between AKI and non-AKI patients and calculated by student-*t* test or Mann-Whitney *U* test, where appropriate. BMI: body mass index, BUN: blood urea nitrogen; AG: albumin to globulin ratio, AST: Aspartate aminotransferase, ALT: Alanine aminotransferase, ALP: Alkaline phosphatase, PT: Prothrombin time: aPTT: activated partial thromboplastin time

We found the development of AKI among dengue patients is significantly associated with higher levels of serum creatinine (*P* < 0.001), BUN (*P* < 0.001), uric acid (*P* < 0.001), alanine aminotransferase (*P* = 0.015), hematocrit (*P* = 0.005) and prothrombin time (*P* < 0.001), when compared with those patients who did not develop AKI. We also observed that patients with AKI had significantly longer hospital stay of 5.79 days than patients without AKI (Table 2).

### Predictors of Acute Kidney Injury in Dengue Infection

**Fig 2:**
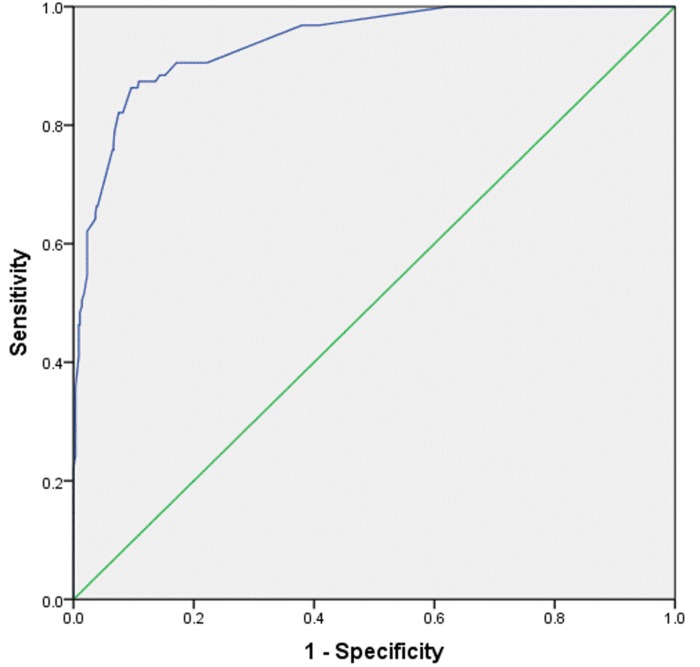
ROC Curve analysis of Multivariate regression model of AKI among dengue patients.

Aiming to determine the factors independently associated with the development of AKI, we developed a series of logistic regression analysis, which are presented in Table 3. Clinically relevant and statistically tested variables were subjected univariate analysis. The variables with *P* values less than 0.25 were considered as candidates for multivariate analysis. The use of univariate *P* values <0.25 has advantage of tending to include more variables in multivariate analysis while traditional levels of *P* value such as 0.05 can fail in identifying variables known to be important [27]. We noted that the factors independently associated with AKI development were male gender (*OR*: 2.7), DHF (*OR*: 8), rhabdomyolosis (*OR*: 7.9), multiple organ dysfunction (*OR*: 17.9), diabetes mellitus (*OR*: 10.5), delayed hospital consultation (*OR*: 2.1) and use of nephrotoxic drugs (*OR*: 2.9). ROC curve analysis with AUC as 0.94 (P<0.001, 95%CI: 0.916–0.964) demonstrated that logistic model has excellent predictive ability for AKI (Fig 2).

**Table 3:** Independent risk factors for AKI by univariate and multivariate analysis.

Variables	Univariate analysis			Multivariate analysis		
	P-value	OR	95% CI for OR	P-value	OR	95% CI for OR
Old age	0.008	3.1	1.34–6.95	0.924	1.1	0.26–4.15
Male Gender	<0.001	8.1	4.79–13.52	**0.012**	**2.7**	**1.25–5.99**
DHF*	<0.001	7.4	4.37–12.57	**<0.001**	**8.0**	**3.64–17.59**
Low albumin	<0.531	2.1	0.90–5.34	–	–	–
Rhabdomyolysis	<0.001	20.7	10.67–40.15	**<0.001**	**7.9**	**3.04–20.49**
Multiple organ dysfunction	<0.001	33.8	19.47–58.76	**<0.001**	**17.9**	**9.14–35.12**
CKD	0.245	1.7	0.71–3.97	0.354	2.2	0.42–11.24
Hypertension	<0.001	5.6	2.78–11.24	0.654	0.8	0.22–2.60
Diabetes Mellitus	<0.001	10.5	5.21–21.34	**0.034**	**4.7**	**1.12–19.86**
Late hospitalization┬	0.009	1.8	1.16–2.823	**0.033**	**2.1**	**1.06–4.13**
Use of nephrotoxic drugs	<0.001	9.1	5.71–14.79	**0.006**	**2.9**	**1.34–6.11**
Elevated ALP	0.770	1.1	0.64–1.85	-	-	-

Variables with P<0.25 (Low albumin, Elevated ALP) were excluded from multivariate analysis

Odds ratio (OR) and Confidence interval (CI) have been rounded off.

DHF: dengue hemorrhagic fever, ALP: alkaline phosphatase, CKD: chronic kidney disease

*All grades of DHF including 5 cases of DSS

┬ patients admitting to hospital on or after day 5 of onset of symptoms

### Outcomes at discharge

All the patients were evaluated for renal and hepatic outcomes at discharge. We found that approximately half of the patients with AKI had renal insufficiencies at discharge. Similarly, hepatic impairments were more prominent among AKI cases (Table 4).

**Table 4:** Patient`s outcomes at discharge.

Outcomes	Overall N = 667	AKI group N = 95	Non-AKI group N = 572	* *P*-value
Fully recovered (without renal and hepatic impairment)	349 (52.3%)	0	349 (61%)	<0.001
Renal functions at discharge				<0.001
Normal renal function	586 (87.9%)	49 (51.6%)	537 (93.9%)	
Renal insufficiency with Scr <200 μmol/L	75 (11.2%)	40 (42.1%)	35 (6.1%)	
Renal insufficiency with Scr >200 μmol/L	6 (0.9%)	6 (6.3%)	0	
Hepatic function at discharge				0.017
Normal hepatic functions	437 (65.1%)	56 (58.9)	381 (66.6%)	
Mild hepatic disturbance	202 (30.3%)	40 (42.1%)	162 (28.3%)	
Moderate to severe hepatic disturbance	38 (5.7%)	9 (9.5%)	29 (5.1%)	
Need of dialysis	0	0	0	-
Need of blood transfusion	39 (5.8%)	7 (7.4%)	32 (5.6%)	0.495

*Pearson Chi-Square

Scr: serum creatinine, μmol/L: micromole per liter

AKI was found to be an independent predictor [OR (95% CI): 1.3 (0.84–2.01), P = 0.044] of longer hospitalization. Moreover, patients with DHF [OR (95% CI): 4.8 (3.13–8.34), P = 0.001], diabetes mellitus [OR (95% CI): 3.4 (2.65–4.53), P = 0.003] and secondary infection [OR (95% CI): 1.7 (1.01–3.61), P = 0.023] were more likely to have renal insufficiencies at discharge in our study.

All the fatal cases had worst AKI and increasing AKI severity was associated with higher mortality where AKIN-II was observed in 6 patients while 2 patients had AKIN-III. Half of died cases had oliguric-AKI while co-morbidities were present in six patients including hypertension in one patient, diabetes mellitus in 2 patients, chronic kidney disease in 2 patients and ischemic heart disease in one patient. These patients were also using nephrotoxic drugs (OR: 4.6, P<0.001) for their pre-morbid conditions. The causes of death in our study were recorded as renal complications, shock and altered mental status in 3, 4 and 1 patients respectively. We found diabetes mellitus [OR (95% CI): 7.1 (5.21–15.32), P = 0.001], DHF [OR (95% CI): 2.9 (1.82–5.11), P = 0.003] and old age [OR (95% CI): 2.2 (1.7–3.18), P = 0.024] as significant independent predictors of mortality. All the fatal cases in our study had AKI therefore logistic regression was not performed to assess the predictive ability of AKI for death.

## Discussion

The current study is a largest single center study comparing all types of dengue infection with regards to AKI. Our analysis stratified patients on the basis of AKIN criteria. The incidence of AKI was 14.2% in our study cohort. Previous investigations have reported incidence of AKI as 13.3% [6] and 10.8% [7] in dengue infection, irrespective of severity, while incidence of AKI among patients with DHF was 0.9% [9], 3.3% [11], 3.9% [13], 9.3% [12] and 12% [8]. Higher incidence in our study might be due to patient selection and referral pattern. Approximately 40% cases were referred from rural areas where medical resources were deficient. Secondly, dengue outbreak occurs in Malaysia every year and study location is one of the major dengue hotspot. Higher incidence of AKI in our study can be due to presence of secondary infection that may lead to severe dengue [28] and severe dengue is well known to be associated with development of AKI [29]. In our study, 20% with AKI had secondary infection. Lastly, higher incidence can be explained by the different selection criteria used to stratify AKI. Laoprasopwattana *et al* [9] and Lee *et al* [11] defined AKI as rapid elevation of serum creatinine levels above 2mg/dL (176.8 μmol/L). This definition might ignore AKIN-I cases which accounted for 76.8% in our study. Khalil *et al* [6] and Mehra *et al* [7] reported the incidence of AKI by using AKIN criteria as13.3% and 10.8% respectively that is comparable with our study. The incidence of AKI among dengue patients has varied significantly across different studies and such difference may be explained by study design, the population studied, differentiation of SCr versus UO criteria, and determination of baseline SCr levels.

Majority of the patients in our study had developed AKI prior to the hospital admission and it might be due to delayed hospitalization among these patients. Moreover, only a minority of patients (1.6%) with no evidence of kidney injury on hospital admission progressed to AKI later. Approximately, 1.6% patients deteriorated into severe AKIN stage during their hospital stay. In-hospital progression of AKI is often associated with longer hospital stay and mortality. Therefore, how to prevent the renal function from deteriorating is crucially important. Low progression rate of AKI in our study might be contributed to vigorous and appropriate hydration among dengue patients. Our study is the first one that demonstrated progression of AKI in dengue patients while closely related previously published literature lack such information [6,7]. Besides these, most of the patients in our study had mild AKI (AKIN-I) that resolved spontaneously during their stay.

Subgroup analysis of adolescents (70 patients) with age 12–18 years showed that all of them had mild dengue infection (i.e. DF) in our study. Out of 70 patients, AKI was observed in 4 cases (5.7%); AKIN-I in three patients and AKIN-II in one patient. It is interesting to note that none of the patient was died in this age group and it might be due to early hospital consultation, absence of co-morbidities and aggressive therapeutic measures in these patients. Likewise these patients were also discharged from hospital within 3 days of admission, suggesting the presence of mild infection. Initially, dengue infection was generally considered to be a pediatric disease but is currently a growing problem in adults throughout the tropics. Our findings also support such shift in age pattern of dengue infection.

Due to complexity of AKI pathogenesis, determination of exact mechanisms appeared rather difficult, even in prospective studies[30]. Several pathophysiological perpetrators for the development of AKI have been proposed. Deposition of immune complex in glomeruli [18], hemolytic uremic syndrome [20], multiorgan failure (MOF), rhabdomyolysis or myositis, and direct viral invasion are some etiopathological mechanisms causing AKI during DVI [21]. We found liver as most affected organ followed by spleen, pancreas, muscles, lungs and heart. Patients with multiple organ dysfunctions (MODs) were associated with 17.9 times odds of developing AKI in our study.

Previous reports suggested that DHF and DSS contribute to development of AKI [9–11, 13]. Our results supported the previous findings. Among 95 AKI patients, 37 (38.9%) had severe dengue (DHF and DSS) and logistic regression demonstrated that patients with severe dengue were more likely to develop AKI (*OR*: 8). DHF is characterized by increase vascular permeability resulting in plasma leakage which leads to development of DSS [31]. Meanwhile, vascular leakage and hemostasis disturbances associated with DSS lead hypoperfusion (hypotension) and hypoxia which in turn causes decreased kidney perfusion and acute tubular necrosis [32, 11]. Hypoperfusion was observed in 28.4% patients with AKI. On the other hand, it has been hypothesized that direct invasion of muscles or over production of myotoxic cytokines by dengue virus (DENV) causes rhabdomyolysis and AKI might be resulted due to accumulation of myoglobin in kidneys [33]. Out of 95 AKI patients, rhabdomyolysis (assumed by elevated CK levels) was present in 34 (35.8%) patients. Additionally, elevated LDH usually indicates acute muscle injury and was observed in 8.4% patients with AKI as compared to 5.2% patients without AKI, though difference was not statistically significant (*P* = 0.231). Based upon these findings, it can be hypothesized that AKI in our patients might be either due to presence of rhabdomyolysis, hemolysis, hypotension, multiple organ dysfunctions or severe dengue (DHF and DSS).

DVI causing coagulation abnormalities as disseminated intravascular coagulation (DIC) has been reported previously [34]. Concomitant prolongation of PT and aPTT in addition thrombocytopenia was observed in 37.4% patients with AKI suggesting potential DIC development in our study. Unfortunately definitive diagnosis for DIC was not done due to unavailability of data i.e. presence of decreased fibrinogen and increased fibrin degradation products. Nevertheless, possibility of DIC development among AKI patients with simultaneous prolongation of aPTT and PT was high in our study.

The higher incidence of AKI in male gender (*OR*: 2.7) can potentially be attributed to increase mobility of male population in our society that put them at a higher risk of being infected with dengue and hence AKI. Additionally, better access to health care facilities and ease of reporting to physicians might be another reason of high incidence of dengue infection and AKI among males.

The use of nephrotoxic drugs was also significantly (*P* < 0.001) higher in patients with AKI than without AKI. Approximately, more than half of the AKI patients were using nephrotoxic drugs for their pre-morbid conditions. These drugs were selective Cox-inhibitors (celecoxib, rofecoxib), naproxen, diuretics, angiotensin converting enzyme inhibitors (ACEIs) and angiotensin receptor blockers (ARBs). These agents impair the critical autoregulation of renal blood flow and result in hemodnamically induced AKI [35]. Our findings suggested that patients using nephrotoxic drugs have 9.1 times more chances to have AKI as compared to non-users. Therefore, optimal measures should be taken to avoid exposure of such drugs among dengue patients.

Unfortunately histophathological studies were not performed in our patients but BUN to creatinine ratio (BCR) < 20 was observed in 73.7% of patients with AKI suggesting intrinsic AKI. It might be due to glomerular injury caused by direct viral invasion and deposition of immune complex in glomeruli. Based upon BCR, pre-renal azotemia was observed in 18.9% of AKI cases and it can be explained by renal hypoperfusion due to dehydration. Urine specific gravity (USG) was not significantly differ between AKI and non-AKI patients although 9.5% AKI patients had USG >1.015 while 38.9% patients had USG <1.015 suggesting presence of pre-renal and intra-renal AKI respectively. These findings suggest hypoperfusion and glomerular injury can be significant etiological factors of AKI in our study. Proteinuria (27.4%) and hematuria (6.3%) were observed in AKI group suggesting glomerulonephritis. The presence of pyuria (9.5%) and urine tubular epithelial cells (8.4%) among AKI patients might be due to tubular interstitial nephritis (TIN) and acute tubular necrosis (ATN) respectively. Histophathological investigations are needed to confirm these assumptions.

We observed that approximately half of the patients in our study were admitted late to the hospital (mean 4.16 days). Among them, 61 (64.2%) patients with AKI and 237 (41.4%) patients without AKI were admitted on day five of onset of symptoms. Guzman et al reported that patients with late hospitalization had advance stage of dengue on admission and were accompanied by rapid deterioration in their clinical conditions [36]. It might be a reason that most of patients in our study had DHF or DSS on admission and none of the patients evolved during hospital stay. Recently, it has also been reported that AKI developed early in the course of dengue with maximum renal function deterioration occurring within the first days [37]. It might be a reason that most of our patients in our study had AKI on admission. Based upon these findings, it can be assumed that late hospitalization may also be a possible contributing factor to increased risk of AKI in our study. We found that patients who were admitted on and after day 5 of illness had 2.1 times more chances to develop AKI. In such cases, early hospital consultation may reduce risks of AKI as well as deterioration of clinical condition. Besides these, early hospitalization among dengue patients may also reduce the risk of mortality and progression into severe dengue [36].

Patients with AKI in our study portended high morbidity and were associated with longer hospital stay (*P*<0.001) resulting in significant burden in terms of cost of care. All patients with AKI had hospital stay ranges from 2 to12 days and duration of hospital stay was proportional to severity of AKI e.g. mean 4.9, 5.6 and 7.1 days in AKIN-I, AKIN-II and AKIN-III respectively. To the best of our knowledge, we did not find any study looking at the impact of AKI severity on hospital stay among dengue patients. These findings suggest that dengue induced AKI not only increase morbidity but also possess financial burden to patients and health care system that is of particular importance in a resource limited settings.

Overall mortality in our study was 1.2% and all died patients had AKI with advanced stage of dengue infection. These patients were admitted on day six of illness with defervesce phase that was followed by rapid deterioration in their clinical conditions. The possible contributing role of underlying co-morbidities commonly seen in these patients especially hypertension and diabetes mellitus is highlighted. These observations coincide with previous reports [38] where acquired co-morbidities were concomitantly present with dengue among died patients. Moreover, old age (>60 years) and use of nephrotoxic drugs were also prominent among fatal cases.

The diagnostic value of UO criteria in the AKI setting has been challenged by Solomon et al [39]. However, higher diagnostic sensitivity of UO than SCr criteria cannot be denied based upon the findings of Macedo et al [40]. We relied on SCr criteria to stratify AKI in our study due to several reasons. First, UO data for each day of hospitalization was not available for all patients. Secondly, we found that there was no significant difference during first 24 hours urine volume in patients with and without AKI. Thirdly, with available data we found concordance between the SCr and UO criteria in our study. The significance of UO criteria to stratify AKI among dengue patients is questionable requiring controlled studies to evaluate its robustness.

Prior to the era of chronic kidney disease (CKD) staging, it was generally accepted that patients who survive an episode of AKI had a ‘good’ renal outcome as assessed by a rapid return of renal function towards baseline values in most patients and by a low incidence of end-stage renal disease (ESRD). Traditionally it was thought that AKI was reversible and, as a consequence, survivors of AKI were not followed up [41]. But Chawla and Kimmel reported that patients, who survive an episode of AKI, might recover adequate renal functions but still such patients are at risk of developing CKD [42]. In our study, up to 42% patients in AKI group had renal insufficiencies and we found the association of DHF, diabetes mellitus and secondary infection with poor renal outcomes among these patients. Post discharge follow-up of these patients is necessary to determine association of AKI with CKD.

No specific preventive strategies are available for AKI apart from adequate fluid resuscitation, management of dengue associated coagulopathies and avoidance of nephrotoxic agents. Inotropic support and noradrenalin are of benefit. Dialysis is certainly beneficial, although the exact of dialysis remains controversial [11].

### Limitations of study

The present study has several limitations, including it retrospective and monocentric design. First, a lack of consensus in the determination of baseline SCr might affect classification and prognosis of AKI. In our study extensive efforts were made to determine baseline SCr levels. Second, we did not investigate exact etiological factors of AKI, as histopathological studies were not performed in our patients. Third, we relied only on SCr criteria to stratify AKI because UO data for each day of hospitalization was not available. Whether the inclusion of UO criteria would have changed the results is not possible to say as it has been documented that patients defined by SCr criteria were more severely ill compared with patients defined with UO criteria [43]. Fourth, only inpatients were included and all the reported values are dependent on the thoroughness of clinician`s documentation. Clinical outcomes of patients may be biased due to lack of standardized management protocol for dengue patients as different management strategies were used. Fifth, all the patients were followed up until discharge and our study lack post-discharge follow-up among AKI survivors. On the other hand, our study is strengthened by large patient population and the comprehensive description of AKI among dengue patients. Additionally preliminary findings of our study have also been presented in International conferences [44, 45].

## Conclusions

In conclusion, in this retrospective study we reported that AKI defined by AKIN criteria had high incidence among dengue patients. In comparison with non-AKI patients, patients with AKI portended significant morbidity, mortality, longer hospital stay and poor renal outcomes, and these are likely to add to the healthcare burden. Presence of male gender, DHF, multiple organ dysfunctions, rhabdomyolysis, diabetes mellitus, use of nephrotoxic drugs and late hospitalization appear to increase incidence of AKI among dengue patients. These findings underscore the need of clinicians’ alertness to this highly morbid and potentially fatal complication of dengue infection. Prior knowledge of expected clinical profile and predictors of AKI development would provide information to identify individuals at higher risk and on the other hand, provide opportunity to clinicians for appropriate management of such patients in timely manners.
