# Correction: Incidence, Characteristics and Risk Factors of Acute Kidney Injury among Dengue Patients: A Retrospective Analysis

**DOI:** 10.1371/journal.pone.0143271

**Published:** 2015-11-13

**Authors:** Tauqeer Hussain Mallhi, Amer Hayat Khan, Azreen Syazril Adnan, Azmi Sarriff, Yusra Habib Khan, Fauziah Jummaat

There is an error in the second sentence of the Results section of the Abstract. The correct sentence is: Presence of dengue hemorrhagic fever [OR (95% CI): 8.0 (3.64–17.59), P<0.001], rhabdomyolysis [OR (95% CI): 7.9 (3.04–20.49)], multiple organ dysfunction OR (95% CI): 17.9 (9.14–35.12), P<0.001], diabetes mellitus [OR (95% CI): 4.7 (1.12–19.86), P = 0.034], late hospitalization [OR (95% CI): 2.1 (1.12–19.86), P = 0.033] and use of nephrotoxic drugs [OR (95% CI): 2.9 (1.12–19.86), P = 0.006] were associated with AKI. Longer hospital stay (>3 days) was also observed among AKI patients (OR = 1.3, P = 0.044).

There is also an error in the definitions section of the Methodology. The correct definition for multiple organ dysfunction should be: multiple organ dysfunction (dysfunction of ≥2 organs, excluding AKI).
